# Massive Solitary Fibrous Tumor of the Hypopharynx

**DOI:** 10.7759/cureus.83739

**Published:** 2025-05-08

**Authors:** David Gaertner, Katharina Stölzel, Felicia Wright, Clara Marie von Bargen, Arne Böttcher

**Affiliations:** 1 Department of Otorhinolaryngology, University Medical Center Hamburg-Eppendorf, Hamburg, DEU; 2 Department of Diagnostic and Interventional Radiology and Nuclear Medicine, University Medical Center Hamburg-Eppendorf, Hamburg, DEU; 3 Institute of Pathology, University Medical Center Hamburg-Eppendorf, Hamburg, DEU

**Keywords:** head and neck neoplasm, hemangiopericytoma, laryngeal tumor, laryngectomy, rare entity, sft, solitary fibrous tumor

## Abstract

We present the case of a 38-year-old female patient diagnosed with a solitary fibrous tumor (SFT) of the right hypopharynx, a rare and atypical localization of this mesenchymal neoplasm. SFT is a rare entity. Most SFTs are benign tumors commonly arising in the pleura, while extrapleural occurrence in locations such as the head and neck region is also well recognized. The aim of this report is to describe this rare clinical presentation, discuss the diagnostic complexities it entails, and emphasize the critical role of complete surgical excision as the mainstay of treatment, as radiation or chemotherapy protocols are not well established or have been proven to be ineffective. Recurrence is mostly associated with incomplete resection, and malignant transformations have been described. Given the challenging anatomic location of the tumor in our patient, a total laryngectomy was required to achieve complete tumor removal.

## Introduction

Solitary fibrous tumors (SFTs) comprise a histologic spectrum of rarely metastasizing fibroblastic mesenchymal neoplasms, including tumors formerly classified as hemangiopericytomas. They were first described by Klemperer and Coleman in 1931, who presented five cases of thoracic manifestation [1]. Although they are commonly thought of as intrathoracic tumors, 50 to 70% of SFTs arise outside the thorax, including in the central nervous system. Once believed to arise from mesothelial cells, these tumors were described using numerous terms such as fibrous mesothelioma, benign mesothelioma, or localized mesothelioma before their occurrence in locations without mesothelial cells was recognized, and hence they were consolidated under the term SFT. The overexpression of CD34 is a common but not specific feature of SFT. Recently, the discovery of the NAB2-STAT6 fusion gene has led to more precise diagnosis of SFT and helped to differentiate between SFT and, for example, gastrointestinal stromal tumors [2]. NAB2-STAT6 fusion variants have also shown prognostic significance since some show higher mitotic count and inferior recurrence-free interval [3]. An estimated 6-18% of all SFTs occur in the head and neck region. Even though they are generally described as benign and slow growing, malignant variants and metastases can occur. Recurrence appears to be related to incomplete excision rather than histopathological grading [4]. Due to the rarity of SFTs in the head and neck region, reporting such cases is essential for improving clinical awareness and guiding management in this anatomically complex region. This case contributes to the limited pool of head and neck SFTs in the literature.

## Case presentation

**Figure 1:**
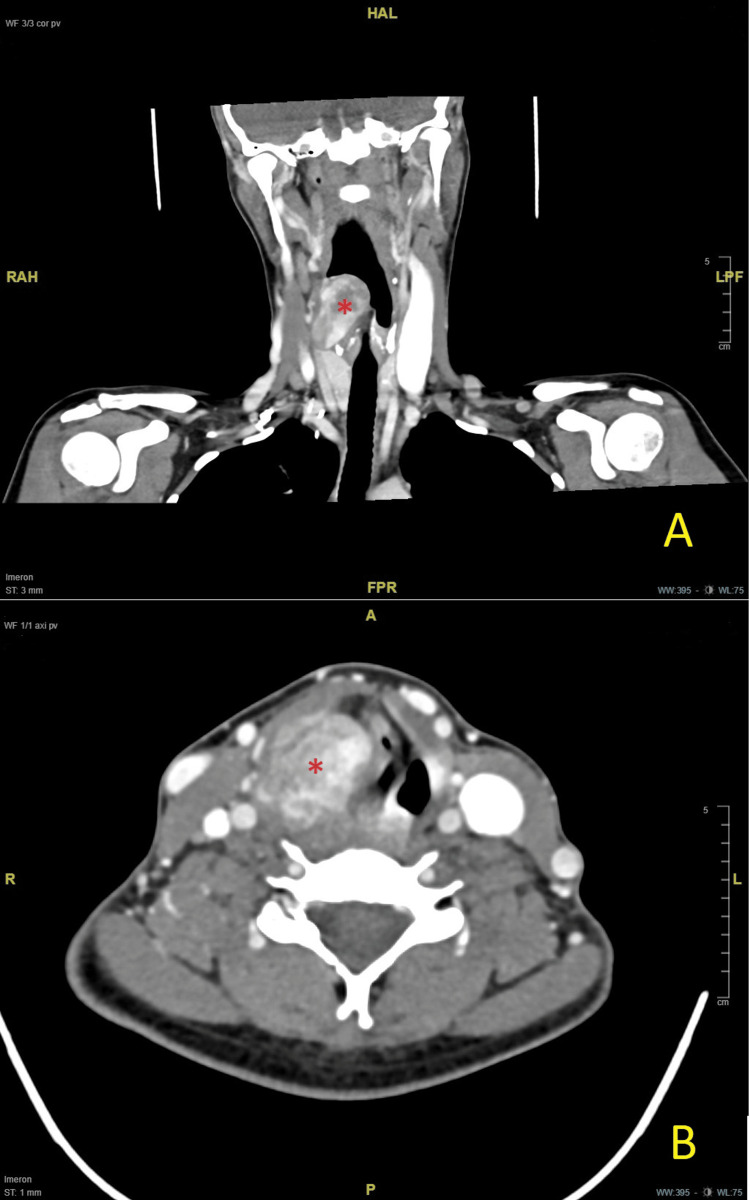
Preoperative CT scans. (A) Coronal view; (B) axial view. *Solitary fibrous tumor attached to the right pyriform sinus, obstructing the larynx.

A 38-year-old woman presented to our emergency room with progressive dyspnea, odynophagia radiating into the right ear, and dysphagia. Food intake was still possible. Dysphonia was absent. The admission laboratory results showed no signs of infection. The clinical examination, including flexible endoscopy, revealed a hypopharyngeal mass on the right side, which was pressing on the laryngeal aditus. The glottis was no longer visible. The patient had no comorbidities or history of excessive alcohol or tobacco use. Computed tomography (CT) revealed an inhomogeneous tumor adjacent to the posterior larynx (Figure 1).

**Figure 2:**
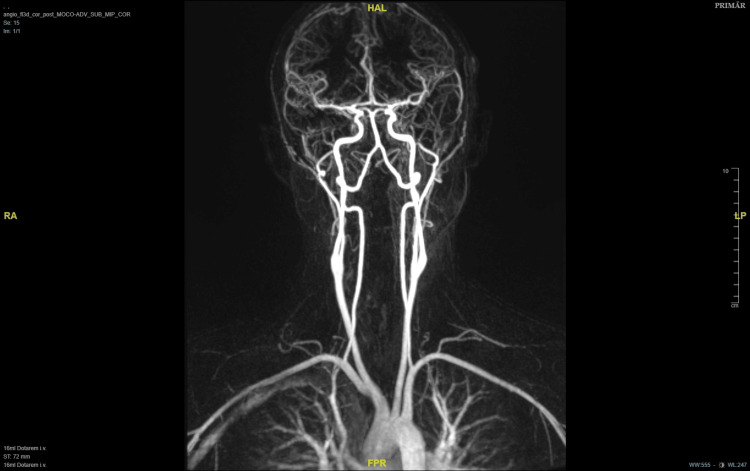
Magnetic resonance angiography in coronal view. There is no arterial hypervascularization of the tumor.

Magnetic resonance angiography showed no signs of arterial hypervascularization of the tumor (Figure 2).

**Figure 3:**
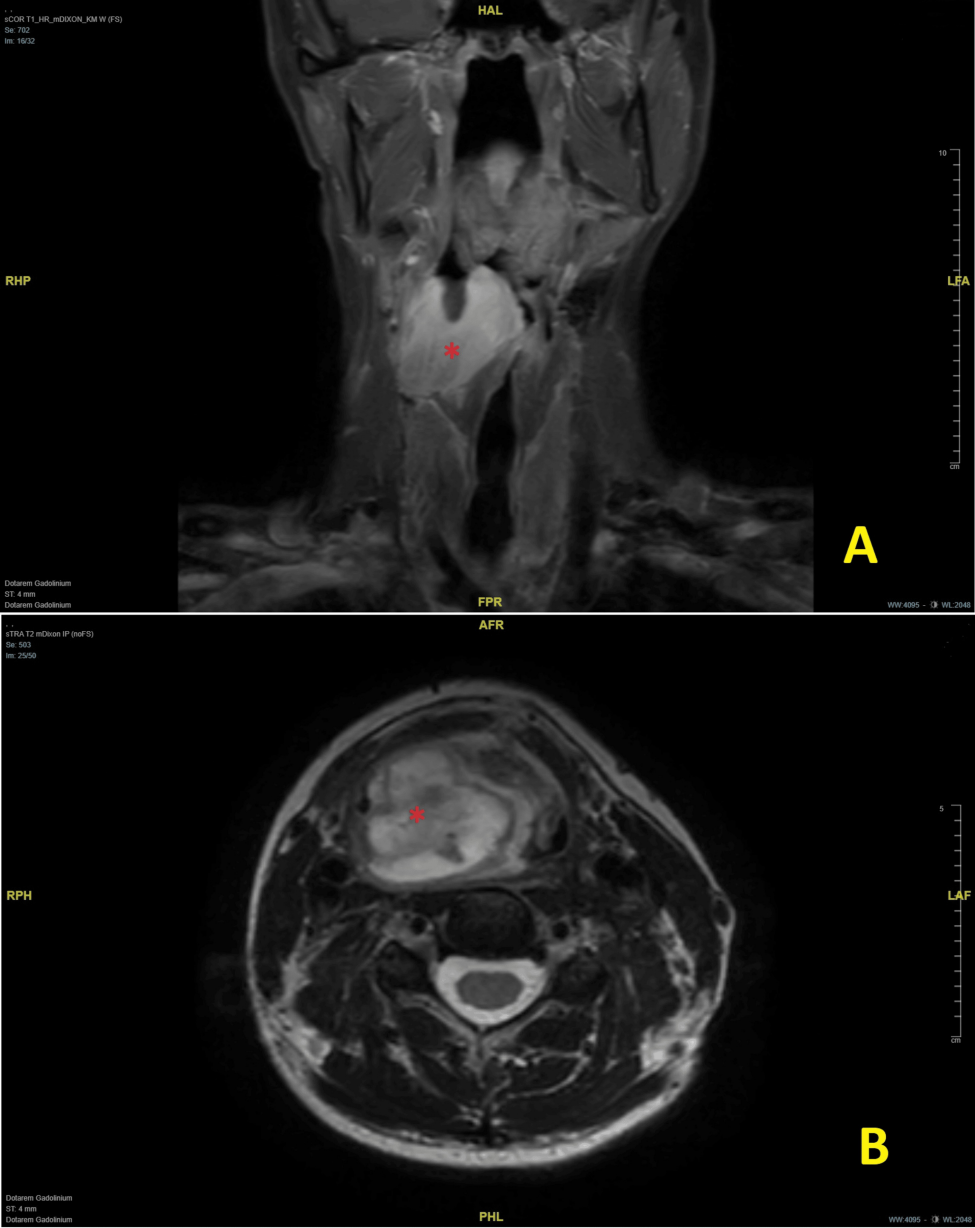
Revised planning MRI. (A) Coronary view, T1 weighted; (B) axial view, T2 weighted. Short-term rapid tumor growth of the partially resected mass (*) in the pyriform sinus.

Tissue samples were obtained via emergency panendoscopy, during which a tumor debulking was performed to prevent the necessity of tracheostomy, prompted by the patient's presentation with progressively worsening dyspnea. Histopathological examination revealed an SFT with a typical haphazard arrangement of spindle cells in a patternless pattern and staghorn branching vasculature, as well as positive STAT6 expression. Following discussion of the case in our head-and-neck tumor and sarcoma board, we initially planned for a trans-oral resection with preservation of the larynx. However, contrary to our expectation, during surgery, the tumor showed a significant size progression beyond the previously described limits, only three weeks after the initial debulking. Therefore, we decided intraoperatively to stop the resection, obtain new deep tissue samples for reanalysis of the mitotic rate, and create a protective tracheostomy. A further magnetic resonance imaging (MRI) scan of the neck was obtained, which showed rapid short-term tumor growth of the partially resected mass at the pyriform sinus on the right side, now with extensive central effusion to the prevertebral region and midline crossing to the left side (Figure 3).

**Figure 4:**
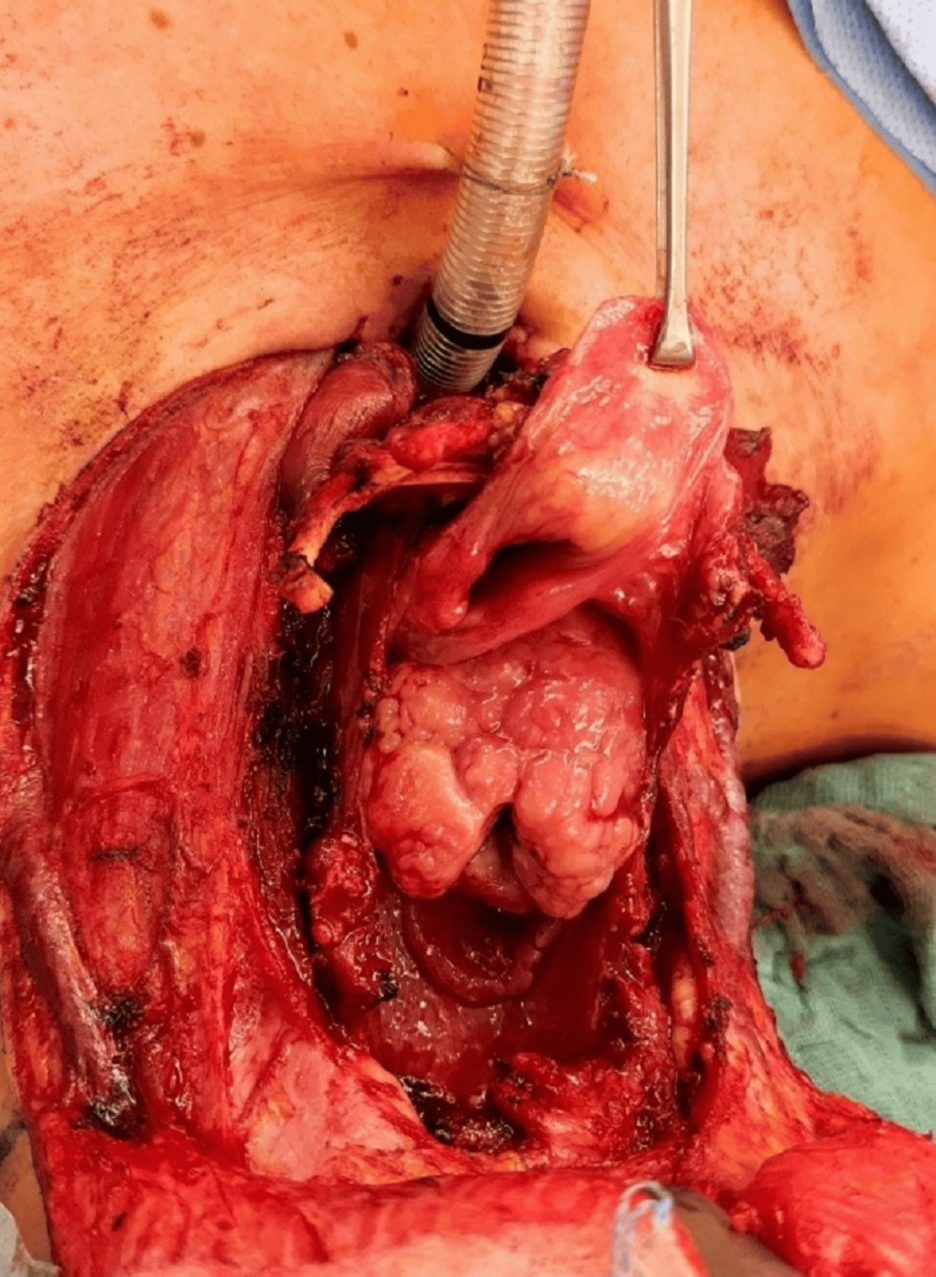
Intraoperative site during laryngopharyngectomy.

**Figure 5:**
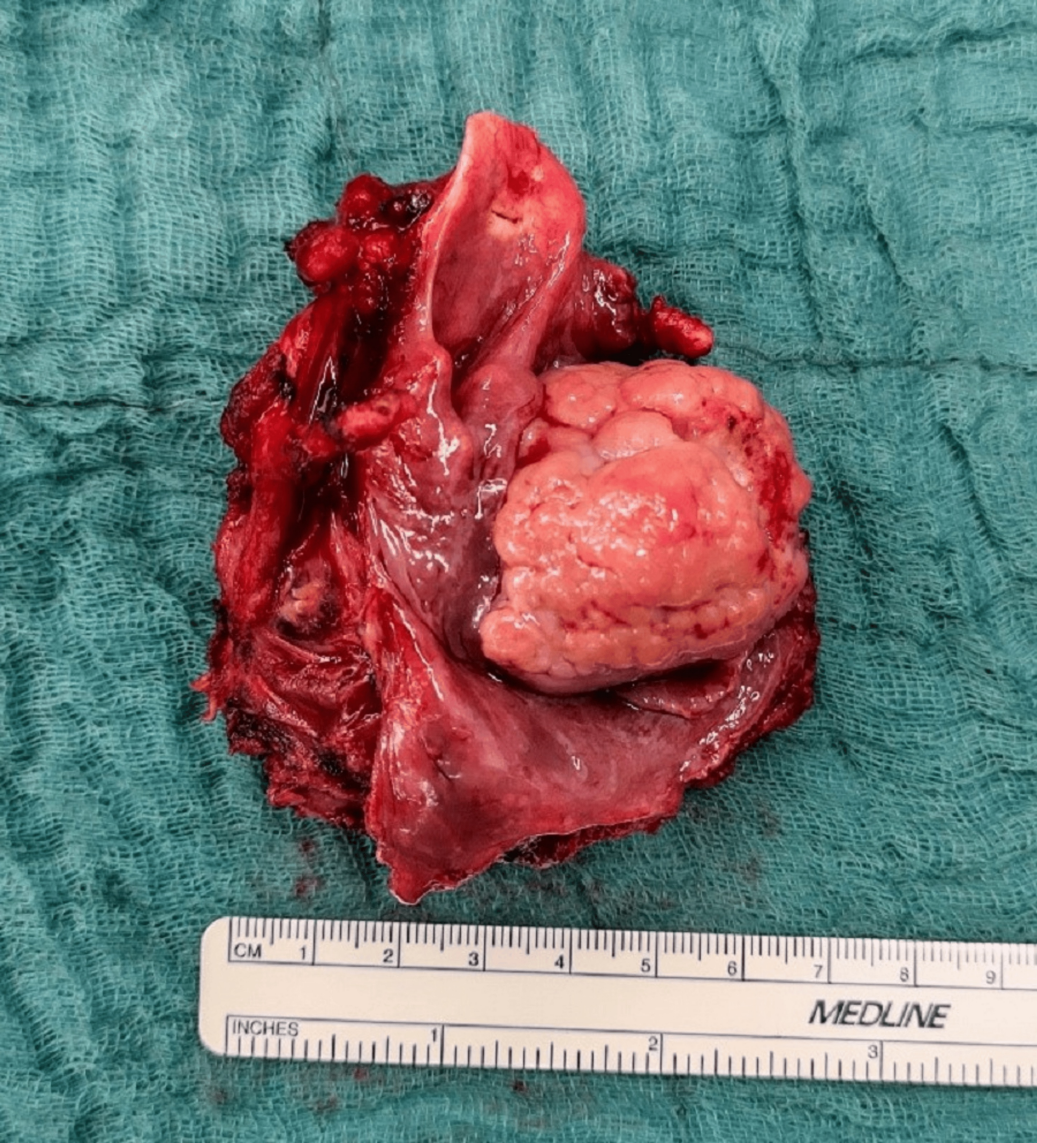
Laryngopharyngectomy specimen ex situ.

Fortunately, histological analysis ruled out malignant transformation, with no mitotic figures or necrosis observed. Due to the rapid progression of the tumor, it was our unanimous consensus that a total laryngopharyngectomy was the only feasible option for the patient, as radiation, chemotherapy, or tumor debulking were unlikely to reinstate the function of the larynx. After consulting with the patient and obtaining her consent, the resection of the tumor was performed six days later (Figure 4 and Figure 5).

**Figure 6:**
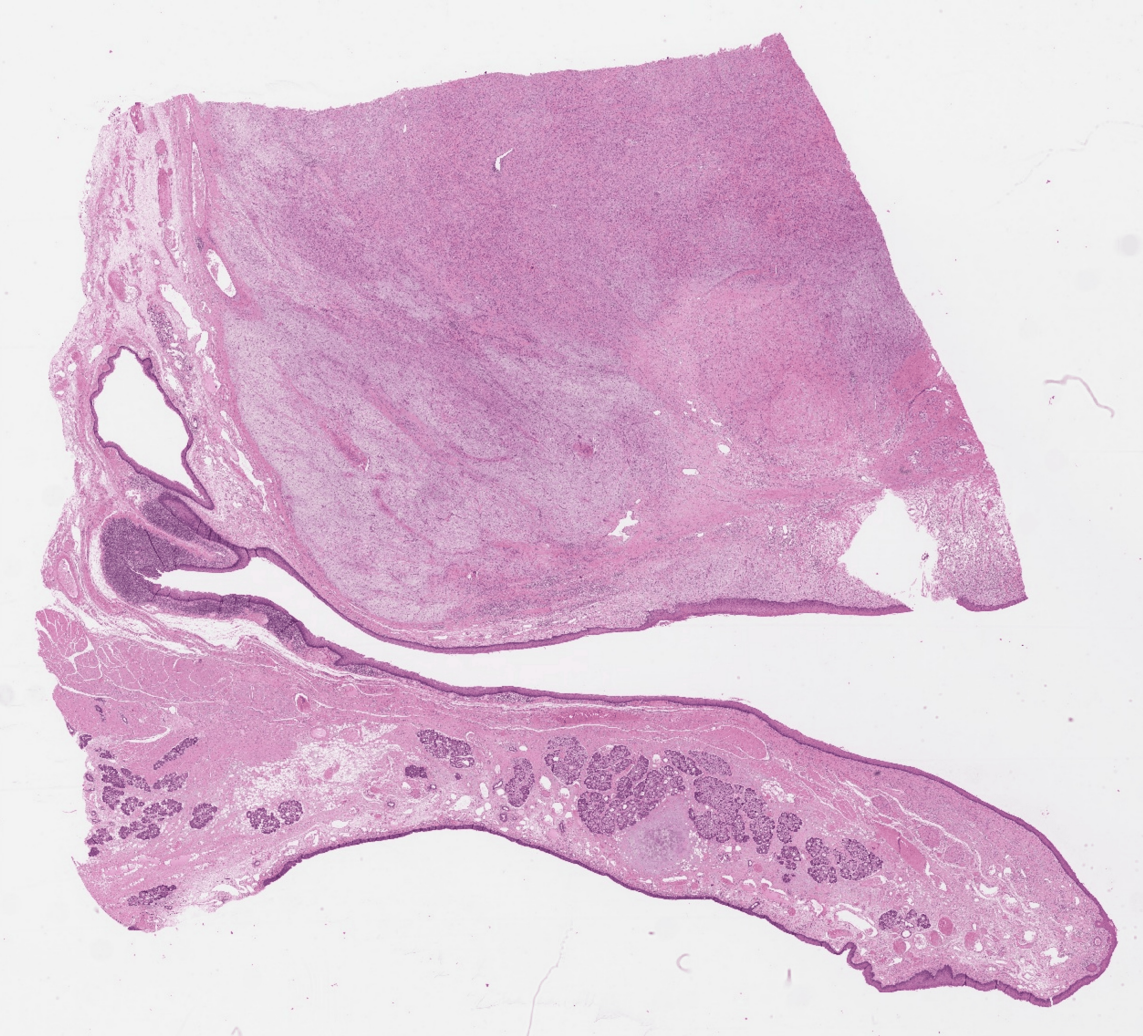
Histological overview shows a well-circumscribed submucosal tumor in the dorsal larynx (H&E staining ×15).

**Figure 7:**
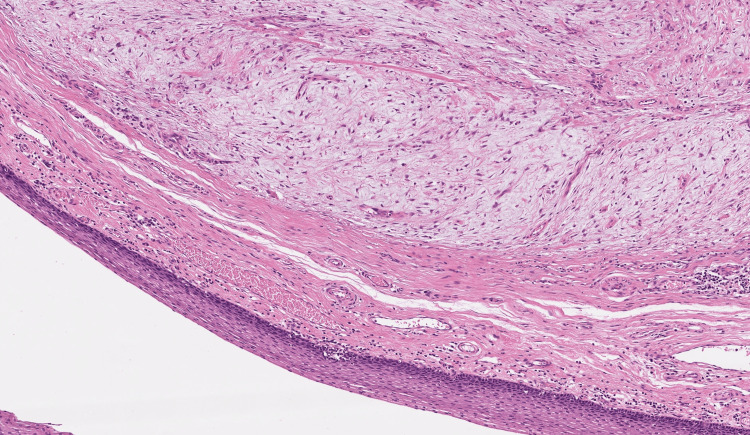
Solitary fibrous tumor (SFT) below regular larynx mucosa (H&E staining ×15).

**Figure 8:**
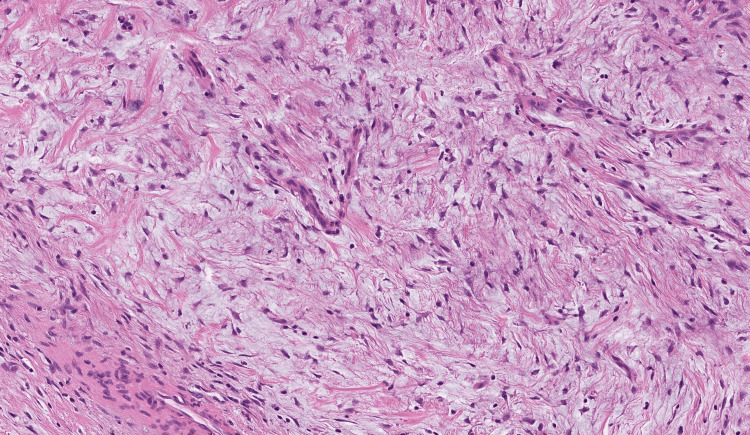
Solitary fibrous tumor (SFT) showing haphazard arrangement of spindle cells with a typical patternless pattern and staghorn branching vasculature (H&E staining ×500).

Due to the massive extension of the tumor, a total laryngopharyngectomy and pharyngeal reconstruction via a tubulated radial forearm flap were conducted. The SFT was excised completely with clear margins and with no postoperative complications. The histology, fortunately, showed no increased risk of malignant transformation (Figures 6-8).

The patient was discharged 20 days after surgery. A follow-up endoscopy two months after the initial surgery revealed two intraluminal masses within the neo-pharynx. A biopsy ruled out a recurrence of the SFT and showed only granulation tissue.

## Discussion

The unfortunate location as well as the rapid growth of the SFT in our patient’s case called for a radical resection via laryngopharyngectomy. This treatment decision should not serve as the standard of care. In our opinion, a mere debulking in this case would have led to a prolonged period of suffering for the patient with a high risk of recurrence and complications such as asphyxiation, aspiration pneumonia, dysphagia, and hoarseness. The decision to take such a radical approach, however, should always be made in a multidisciplinary consensus, as in our case. Xu et al. published a case report of a patient who had no treatment for three years before he underwent surgery. They observed a tumor volume doubling time of 350 days initially, which accelerated to 180 days over the course of time [5]. The authors have no information on a possible correlation between an increase in tumor growth and previous tissue sampling. The increase in growth rates appears to occur spontaneously. Given the considerable size of our patient’s tumor, approximately 4 × 3 × 3 cm at initial consultation, an advanced stage of the disease had to be considered, so a watch-and-wait approach after debulking seemed not to be an option. In addition, there are several case reports of malignant transformation in recurrent disease [6]. Demicco et al. developed a risk stratification model for metastasis based on age, tumor size, and mitotic rate [7]. According to this model, our patient was at low risk of metastasis, with tumor size above 5 cm being the only positive risk factor. Other treatment options, such as radiation or chemotherapy, are not currently recommended for SFTs, as data and established treatment protocols are lacking, given the rarity of this tumor. The role of adjuvant radiation is also unclear, and case reports are mainly available for patients with metastatic disease. A recently published review failed to show a significant benefit of adjuvant radiotherapy on survival. In addition to the skull base serving as the origin site, insurance status was shown to be associated with the highest risk for long-term survival, suggesting a lack of follow-up as the cause [8]. Several studies have tried to establish chemotherapy protocols, but the available data for doxorubicin-based regimens, temozolomide, and bevacizumab or antiangiogenics showed poor results, with a partial response at best [2]. Smith et al. [9] observed local recurrence up to 228 months after resection. We will therefore include the patient in long-term follow-up care, as recommended by many other studies [10,11]. To date, 1.5 years after her initial presentation, our patient is recurrence-free.

## Conclusions

SFTs are challenging entities to treat. The therapeutic options for this disease are based on case reports and can only be considered to a limited extent due to the heterogeneous sites of manifestation. Further studies on treatment are necessary. However, in this case, complete resection with clear margins via laryngopharyngectomy allowed for long-term disease-free survival.
